# Supplementary material for: A Primer on the oxDNA Model of DNA: When to Use it, How to Simulate it and How to Interpret the Results
Source: Front Mol Biosci. 2021 Jun 17;8:693710. doi: 10.3389/fmolb.2021.693710 (PMC8256390; doi:10.3389/fmolb.2021.693710)
Supplement: Supplementary file 1 [file DataSheet1.pdf]

Supplementary Material

1 REDUCED UNITS IN OXDNA

Input and output files use a specific set of reduced units (often called Lennard-Jones units) to represent the system. These units are referred to as “simulation units” in this review. Both the standalone implementation and the LAMMPS module use the same reduced units for lengths and energies.

- In the model, one unit of length σ corresponds to 8.518 Å. This value was chosen to give a rise per bp of approximately 3.4 Å and equates roughly to the diameter d of a nucleotide.
- One unit of energy is equal to ε = 4.142$\times10^{-20}$ J (or equivalently, k_BT at T = 300 K corresponds to 0.1ε).
- The cutoff for treating two bases as “stacked” or “base-paired” is conventionally taken to be when the relevant interaction term is more negative than -0.1 in simulation units (4.142$\times10^{-21}$ J).
- The units of energy and length imply a simulation unit of force equal to 4.863$\times10^{-11}$ N.

For the purpose of dynamical simulations, it is necessary to define a simulation unit of mass. For historic reasons, the standalone code and the LAMMPS code use slightly different definitions.

In the LAMMPS code,

- 1 simulation unit of mass M is taken to be 100 AMU or 1.661×10^{-25} kg with a mass of $M = 3.1575$ per nucleotide in simulation units.
- When combined with the simulation units for energy ϵ and length σ, the mass unit M implies a simulation unit of time $\tau = \sigma\sqrt{M/\varepsilon}$ of 1.706 ps.

In the standalone code,

- 1 simulation unit of mass M is 5.243×10^{-25} kg and nucleotides are assumed to have a mass $M = 1$ in simulation units.
- The simulation unit of time τ is therefore 3.031 ps.

It is worth noting that both approaches treat the nucleotide as a sphere for the purpose of evaluating its equation of motion. The LAMMPS code uses a moment of inertia of 0.435179 in simulation units; the standalone code uses 1 in simulation units.

In dynamical simulations, it is also necessary to set the diffusion coefficient D or the mobility μ. For a sphere, both are related through the Einstein-Smoluchowski equation $D = \mu\, k_B\, T$, whereas the mobility is given through the Stokes-Einstein relation $\mu = 1/(3\pi\, \eta\, d)$ with η as solvent viscosity and d the diameter. Typical values for aqueous solutions are $\eta = 10^{-2}$ Poise $= 10^{-3}$ kg m^{-1}s^{-1} or 8.749 $M\sigma^{-1}\tau^{-1}$ in LAMMPS simulation units.

It is instructive to determine the inertial and Brownian timescale from these values. The inertial timescale $\tau_{in} = 1/\gamma$ is the timescale on which momentum relaxation occurs, and is the inverse of the friction constant $\gamma = 1/(m\mu)$ with m as mass of the inert object. This leads to $\tau_{in} = 3.829 \times 10^{-2}\tau$ in LAMMPS simulation units. The Brownian timescale $\tau_B = d^2/D$ is the time it takes an object undergoing Brownian motion to diffuse over its own diameter. At $T = 300$ K we obtain $\tau_B = 8.246 \times 10^2$ τ, therefore

$\tau_{in} \simeq 4.644 \times 10^{-5}\tau_B \ll \tau_B$, which means both timescales are separated by more than five orders of magnitude.

To improve the sampling efficiency and to maximise the actual physical time of a simulation, it is well justified to speed up the diffusion and opt for larger simulation values of D and μ provided the sequence of timescales is not violated. This means $\tau_{in} = 1/\gamma < d^2\, m\,\gamma/k_BT = d^2\, m/(k_BT\tau_{in}) = \tau_B$. In LAMMPS units this leads to $\tau_{in} < \sqrt{3.1575/k_BT}$ or $\gamma > \sqrt{k_BT/3.1575}$, so for instance at $T = 300$ K $\tau_{in} < 5.619$ in simulation units. Setting $\tau_{in} = 2.5$ entails $\tau_B \simeq 5\,\tau_{in}$ and results in inertial and Brownian timescales that are sufficiently separated.

2 ACCURACY OF THERMOSTATS

MD simulations only sample from the correct Boltzmann distribution in the limit of small integration timesteps. In this section, we illustrate the performance of the integrators in terms of reproducing the correct average energies. For the purposes of these simulations, we consider a small duplex with an overhanging single-stranded tail, simulated using oxDNA1.0 at 300 K. Sequences:

- 3'-TTTTTGACTTGGA-5'
- 3'-TCCAAGTC-5'

Simulations in the standalone model were performed by setting the parameter "diff_coeff" to 2.5, implying a diffusion coefficient of single particles of 2.5 simulation units. The simulations are performed at 300K with number of timesteps for the 4 MD simulations being 3.5×10^7, 1.1×10^7, 7×10^6, 5×10^6 for $\Delta t = 0.001, 0.003, 0.005$ and 0.007 respectively. For these MD simulations, a simple thermostat that emulates Brownian dynamics is used. Velocity and angular momentum of the particles are refreshed (or at least attempted to) every 103 time steps. For the VMMC simulations, a total of 1×10^7 steps are attempted per particle. Each set of simulation is run 5 times with different initial configurations. Energy values, as shown in Table S1, are then averaged over these 5 simulation runs. For the MD simulations, energy values of the configurations are output at every 1,000 timesteps for the 4 different Δt values. For the VMMC simulations, energy values are output after every 1,000 steps per particle.

Simulation in LAMMPS were performed by setting the inertial timescale $\tau_{in} = 1/\gamma$=2.5, which is controlled via the "damp" parameter in the Langevin thermostat. This corresponds to a diffusion coefficient $D = k_BT/m\gamma = 0.07918$ in LAMMPS simulation units. We equilibrated the benchmark for 5×10^6 iteration steps using a timestep size $\Delta t = 0.01$ and temperature $T = 0.1$. The equilibrated configuration was used as initial configuration for 10 independent runs with different random seeds and timestep sizes Δt. For each timestep size we adjusted the number of iteration steps so that the runs were performed over the same simulation time $t = 100,000$ (e.g. 10^7 iteration steps for $\Delta t = 0.01$ and 10^8 iteration steps for $\Delta t = 0.001$. The output frequency was adjusted in the same way to have an energy reading every $t = 0.1$. Each set of 10 runs was then averaged. The results are given in Table S2.

Δt	PE	KE
0.001	-1.265(2)	0.2999(3)
0.003	-1.262(5)	0.3004(11)
0.005	-1.263(3)	0.3014(4)
0.007	-1.237(4)	0.3067(6)
VMMC	-1.2658(5)	-

Table S1. Mean potential and kinetic energies per nucleotide for MD simulations in the standalone code (using the units of the standalone code). The results for the potential energy in VMMC simulations are included for comparison. Brackets give standard error on the mean of final digit(s).

Δt	PE	KE
0.001	-1.242(3)	0.30000(4)
0.003	-1.240(2)	0.29996(2)
0.005	-1.239(3)	0.30003(1)
0.007	-1.241(2)	0.30004(2)
0.01	-1.245(1)	0.30006(2)
VMMC	-1.2658(5)	-

Table S2. Mean potential and kinetic energies per nucleotide for MD simulations using the Langevin thermostat in the LAMMPS code ("damp" set to 2.5, using the units of the LAMMPS code). The results for the potential energy in VMMC simulations (performed in the standalone code) are included for comparison. Brackets give standard error on the mean of final digit(s).

In Tables S1 and S2, the VMMC results for the potential energy are assumed to be accurate (to within sampling noise) since, unlike the MD algorithms, VMMC does not require a limit to be taken before its stationary distribution converges on $p_{\mathrm{eq}}(x)$. For the kinetic energy, equipartition gives the correct value as 0.3 in oxDNA units (kT is 0.1, and each nucleotide has six momentum degrees of freedom)

Overall, for the standalone integrator performance has substantially worsened by $\Delta t = 0.007$. $\Delta t = 0.005$ performs reasonably, but it should be noted that stability issues sometimes arise when that time step is used in GPU simulations, perhaps due to extra errors introduced by the use of mixed precision. Generally, we would recommend $\Delta t = 0.003$ as safe for this (typical) choice of "diff_coeff".

The Langevin integrator shows no noticeable degradation in performance over the range of timesteps. However, the results do show a systematic offset in the potential energy which we have attributed to a minor problem in the LAMMPS implementation that leads occasionally and locally to slightly frustrated stacking interactions in very flexible ssDNA overhangs, such as those occurring in the benchmark for the energy comparison runs. We are going to fix this issue in the next stable LAMMPS release scheduled for summer 2021 (contact: O.H., oliver.henrich@strath.ac.uk). Nonetheless, the results suggest that when this problem is fixed, simulations of up to timestep of $\Delta t = 0.01$ are feasible (at least for this value of the "damp" parameter). For larger values of "damp" (actually equating to weaker damping), smaller timesteps are necessary to prevent instability.
